# Understanding CT Resolution Timeline in Acute Pyelonephritis: A Framework for Clinical Practice

**DOI:** 10.7759/cureus.115775

**Published:** 2026-09-04

**Authors:** Steven S Chua, Leela Chaudhary, Blaine T Abraha, Jiries Ganim, Yasmin A Toy, Areeba Hafeez, Joseph P Hasapes, Gabriel M Aisenberg

**Affiliations:** 1 Radiology, University of Texas Health Science Center at Houston, Houston, USA; 2 Internal Medicine, University of Texas Health Science Center at Houston, Houston, USA

**Keywords:** computed tomography, diagnostic imaging, perinephric fat stranding, pyelonephritis, sensitivity and specificity

## Abstract

Background

Acute pyelonephritis is diagnosed based on clinical and laboratory signs. Computed tomography (CT) is typically reserved for detecting complications or alternative diagnoses. Current guidelines do not recommend routine follow-up imaging when abnormalities are found. The expected time to resolution (TTR) of CT abnormalities has not been clearly identified, which serves as the goal of our study.

Methodology

This retrospective study was conducted at an academic hospital in Harris County, Texas. Adult patients with a diagnosis of acute pyelonephritis who underwent at least two CT scans were included. Three board-certified radiologists independently reviewed CT studies performed within one year of the index scan and assessed for striated nephrogram, parenchymal edema, patchy hypodensities, perinephric fat stranding, and urothelial thickening. Time to first documented radiologic resolution (TTR) was defined as the interval between the first positive CT sign and the first subsequent CT in which the sign was absent. TTR was calculated for individual signs and globally, provided clinical criteria for pyelonephritis were met. Sensitivity, specificity, and inter-rater agreement were also evaluated.

Results

A total of 288 CT scans from 93 patients were reviewed, of which 252 (88%) were of sufficient quality for analysis. The mean global TTR was 149 ± 100 days, with wide variation across individual signs. Moreover, imaging abnormalities persisted for 105 days despite clinical resolution.

Conclusions

CT signs consistent with pyelonephritis may persist for several months after clinical recovery. Clinicians should interpret persistent imaging abnormalities appropriately to avoid unnecessary diagnostic or therapeutic interventions.

## Introduction

Acute pyelonephritis is an infection of the renal parenchyma, renal pelvis, and calyces. The diagnosis is primarily clinical and is based on the presence of fever, flank or lumbar pain, costovertebral angle tenderness, and pyuria. Computed tomography (CT) is generally reserved for evaluating complications or associated features, such as gas formation, hemorrhage, urinary tract obstruction, nephrolithiasis, or abscess formation [1,2]. Characteristic CT signs in cases of pyelonephritis include a striated nephrogram, parenchymal edema, patchy hypodense areas, perinephric fat stranding, and smooth muscle urothelial thickening [3-8].

Magnetic resonance imaging (MRI) provides an alternative imaging modality, but its higher cost, longer scan times, claustrophobia, and limited availability restrict routine use. Current recommendations from the American College of Radiology do not advise routine follow-up imaging after an episode of uncomplicated acute pyelonephritis [9]. Nevertheless, patients frequently undergo subsequent abdominal imaging for persistent symptoms, evaluation of complications, recurrent urinary tract infections, or unrelated clinical indications. In these situations, residual renal abnormalities may be encountered and can be difficult to distinguish from active infection. Knowledge of the expected duration of CT abnormalities may therefore help clinicians avoid unnecessary diagnostic testing, prolonged antimicrobial therapy, or additional imaging studies. However, current guidelines do not address the expected timeline for radiologic resolution or the duration of imaging abnormalities.

The objective of this study was to determine, in patients with both clinical and radiological signs consistent with pyelonephritis, the time to radiologic resolution (TTR) on CT. Specifically, we sought to characterize the persistence of individual CT findings after the initial episode of pyelonephritis and to compare their duration with the timing of clinical recovery.

An earlier version of this manuscript was posted to the Research Square preprint server on April 27, 2026 (doi: 10.21203/rs.3.rs-9390703/v1).

## Materials and methods

This retrospective study was conducted at Lyndon B. Johnson Hospital, a safety-net academic hospital in Harris County, Texas, with previous approval by the Committee for the Protection of Human Subjects (approval number: HSC-MS-25-0660). Among approximately 48,000 CT scans performed between January 1, 2023, and December 31, 2023, about 1,000 reports described signs consistent with acute pyelonephritis.

Patients aged 18 years or older with International Classification of Diseases, Tenth Revision (ICD-10) code N10 for acute pyelonephritis [10] were screened for inclusion using the institution-wide electronic medical record database if they had undergone at least two CT scans, with the first scan obtained during the study period.

Clinical information necessary to confirm cases of acute pyelonephritis was extracted independently from the radiologic review process by study investigators who were not involved in image interpretation. Data included fever, flank or lumbar pain, costovertebral angle tenderness, pyuria, urine culture results when available, indications for CT examination, and documented clinical assessment at the time of each encounter. Radiologists were not provided access to these clinical data during image review.

Eligible cases were independently reviewed by three board-certified radiologists who had completed training 9 to 30 years earlier; the radiologists assessed CT scans obtained within one year of the index study. For each scan, the radiologists recorded the presence or absence of the following signs (Figure 1): striated nephrogram, parenchymal edema, patchy hypodensities, perinephric fat stranding, and smooth urothelial thickening.

**Figure 1:**
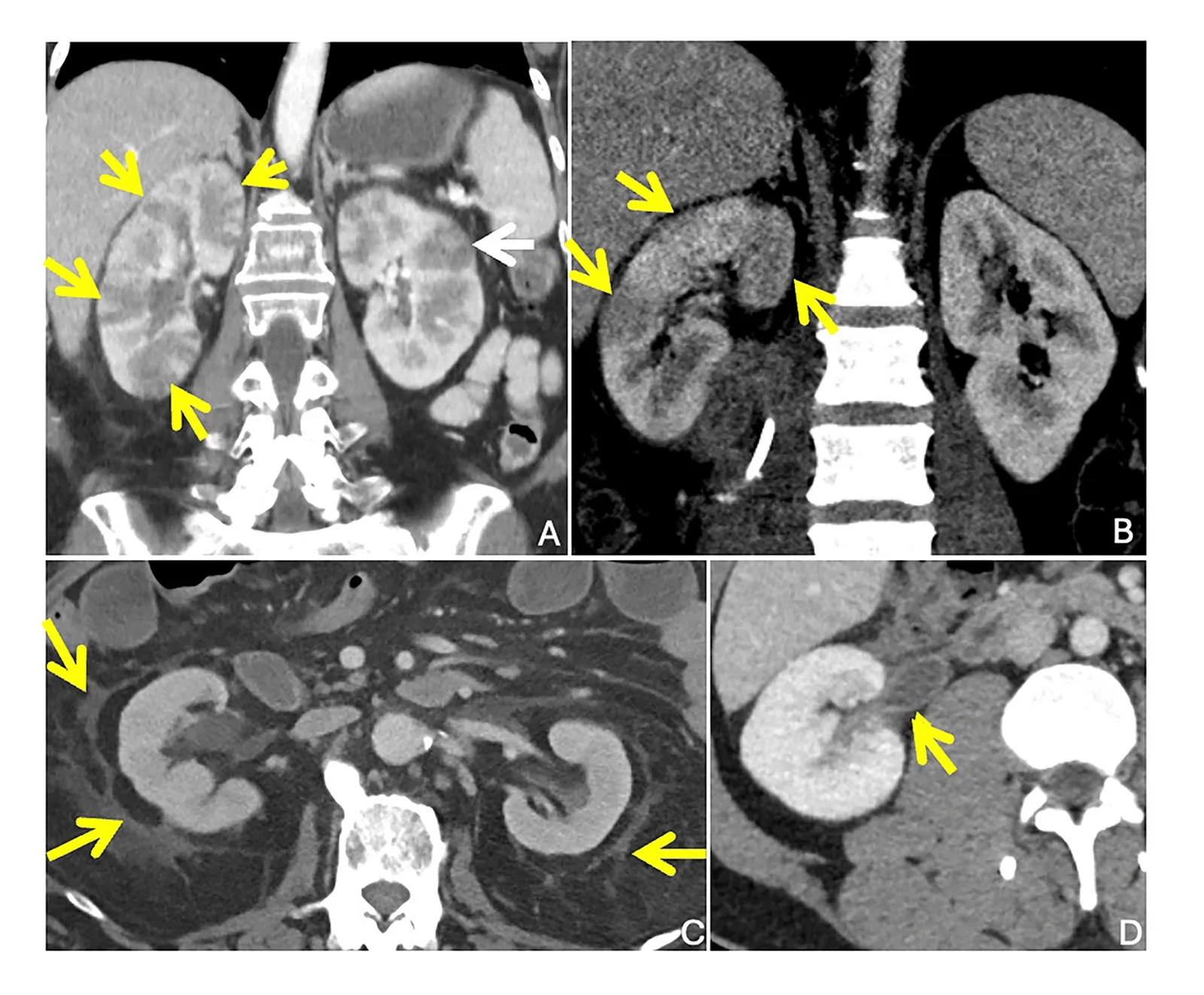
Radiologic signs explored among the included examinations. (A) Striated nephrogram, with areas of radiating hypodense and hyperdense wedge regions (yellow arrows) of the right kidney. This can be seen in pyelonephritis and obstructive uropathy. Edema and wedge-shaped patchy hypodensity of the left kidney (white arrow). (B) Right kidney with areas of peripheral wedge-shaped patchy hypodensities with edema (arrows). The left kidney is normally enhanced. (C) Bilateral perinephric stranding (yellow arrows) of the kidneys with trace perinephric fluid. (D) Urothelial thickening and enhancement of the right renal collecting system and proximal ureter (yellow arrow).

Using these data, we calculated time to the first documented radiologic resolution (TTR), both globally (overall resolution of pyelonephritis signs) and individually for each radiologic sign on subsequent CT scans. The CT examinations belonging to a given patient were reviewed sequentially in chronological order. Reviewers were aware that the studies belonged to the same patient, which facilitated the determination of radiologic resolution. While this approach may have introduced expectation bias regarding subsequent examinations, it was considered necessary to establish the temporal evolution of imaging findings.

Definitions

A clinical case of pyelonephritis was defined as the presence of pyuria plus at least one of the following: fever, flank or lumbar pain, or costovertebral angle tenderness, regardless of the results of the urine culture or antibiotic exposure. Clinical resolution represented cases in which fever, flank or lumbar pain, and costovertebral angle tenderness had resolved. Residual pyuria was not considered sufficient evidence of persistent pyelonephritis because sterile pyuria may persist after treatment and may occur in the absence of active renal infection [11].

A true positive was defined as the presence of a positive radiologic sign in a patient meeting clinical criteria for pyelonephritis; a true negative was the absence of a radiologic sign in a patient who did not meet clinical criteria; a false positive was the presence of a radiologic sign in a patient without clinical pyelonephritis; and a false negative was the absence of a radiologic sign in a patient with clinical pyelonephritis.

A striated nephrogram represents an appearance of discrete rays of alternating hypoattenuation and hyperattenuation radiating from the papilla to the cortex along the direction of the excretory tubules. This appearance is attributed to the decreased flow of contrast due to stasis in the infected tubules. This sign is not specific and is also seen in some other conditions, such as renal vein thrombosis, ureteric obstruction, and contusion [12].

Parenchymal edema describes a focal or diffuse renal parenchymal enlargement with decreased attenuation relative to adjacent normal renal cortex and associated loss of corticomedullary differentiation [9].

Patchy hypodensities are defined as focal, ill-defined areas of decreased enhancement within the renal parenchyma relative to the surrounding cortex during the nephrographic phase [9].

Perinephric fat stranding refers to increased attenuation and linear streaking within the perirenal fat on CT, reflecting inflammation, edema, or fluid within the perirenal space. It occurs because inflammatory processes around the kidney increase vascular permeability and fat edema, producing the striated appearance in the surrounding fat. It carries reportedly low specificity, as it can be seen in pyelonephritis, renal or perinephric abscesses, ureteral obstructions, nephrolithiasis, renal infarction, renal vein thrombosis, renal contusion, perirenal hematoma, colonic or pancreatic inflammation involving the retroperitoneum, or after nephrostomy tube placement or renal biopsies [9,13]. Urothelial smooth muscle thickening is usually described in the renal pelvis or ureter.

To be included in the TTR calculation, a given true-positive radiologic sign had to be present on an initial CT and subsequently absent on follow-up imaging. The interval between the first positive and the first negative scan was recorded, provided that clinical and laboratory data supported the diagnosis of pyelonephritis. The same approach was used for global radiologic resolution.

Patients were excluded from TTR analyses if they did not meet the clinical criteria for pyelonephritis or if imaging signs persisted without resolution on subsequent CT scans. We also excluded patients younger than 18 years old. We censored the cases without clinical pyelonephritis and without CT resolution, as we could not ascertain if resolution might have been achieved beyond a one-year follow-up.

Statistical analysis

We used MedCalc version 12.3.0 (MedCalc Software, Mariakerke, Belgium) for statistical analysis. The correlation coefficient (Fleiss kappa) was calculated for each variable analyzed by the participating radiologists [14]. If a study was considered of poor quality and therefore not read by one radiologist but read by the other two, the inter-rater agreement for such a case was calculated for those two radiologists and averaged. If only one radiologist deemed a study readable, then the individual assessment was entered in the analysis. Sensitivity and specificity of each sign were calculated as previously described [15]. A formal control cohort without pyelonephritis was not included. Instead, specificity calculations were derived from encounters that did not satisfy predefined clinical criteria for pyelonephritis. Because resolution could only be documented at the time of follow-up CT examinations, calculated TTR values should be interpreted as estimates of the earliest documented radiologic resolution rather than the precise biological timing of resolution.

## Results

A total of 93 patients met the inclusion criteria, of whom 26 (28%) were male. The mean age (±standard deviation) was 49 ± 14 years. Of the 288 initial CTs analyzed, 252 (88%) were of sufficient quality to evaluate the predefined radiologic signs. All patients had at least two CT scans; 51 underwent a third scan, 29 a fourth, 17 a fifth, and 5 a sixth. Of the 288 encounters, 156 (54%) satisfied the predefined clinical criteria for pyelonephritis. The remaining encounters represented follow-up examinations in which clinical criteria were no longer present, or the CT scans were performed for alternative indications. Common indications for follow-up imaging included persistent symptoms, concern for complications, recurrent urinary tract symptoms, surveillance of previously identified abnormalities, and unrelated abdominal complaints.

Table 1 presents the interrater agreement among the three radiologists for the CT signs evaluated. Perinephric fat stranding, a nonspecific finding recognized both by our radiologists and in the literature, showed the highest level of agreement.

**Table 1:** Inter-rater agreement (Fleiss kappa) for the different signs assessed by the three participating radiologists.

Sign	Kappa	Interpretation
Striated nephrogram	0.29	Fair
Parenchymal edema	0.19	Slight
Patchy hypodensities	0.33	Fair
Perinephric fat stranding	0.5	Moderate
Smooth urothelial thickening	0.33	Fair
Global assessment	0.34	Fair

Table 2 summarizes the TTR of the individual and global signs consistent with pyelonephritis, along with their sensitivity and specificity. On average, CT signs resolved after 149 ± 100 days. Among individual features, parenchymal edema (133 ± 37 days), patchy hypodensities (134 ± 98 days), and perinephric fat stranding (155 ± 114 days) most closely approximated the TTR of the global assessment. In our series, perinephric fat stranding also showed the strongest correlation with the global assessment, and sensitivity and specificity were closest to it.

**Table 2:** Time to resolution of individual and global signs consistent with pyelonephritis.

	Striated nephrogram	Parenchymal edema	Patchy hypodensities	Perinephric fat stranding	Smooth urothelial thickening	Global assessment
True positives	45	33	35	34	35	49
Mean (days)	112	133	134	155	101	149
Median (days)	95	100	113	146	89	136
Standard deviation (days)	41	37	98	114	92	100
Range (days)	6 to 361	6 to 364	6 to 361	18 to 364	6 to 269	6 to 361
Sensitivity	42%	33%	48%	65%	43%	67%
Specificity	79%	89%	59%	28%	59%	42%

Table 3 details the number of studies in which signs persisted on follow-up CT scans and the duration of these signs in patients with and without clinical pyelonephritis. Imaging abnormalities tended to persist longer in patients with clinical pyelonephritis; however, we could not determine whether clinical resolution occurred between episodes. In patients without clinical pyelonephritis, abnormalities persisted for a shorter period than that observed in the TTR analysis. Because imaging follow-up did not extend beyond one year, these cases were censored.

**Table 3:** Time persistence of unresolved signs in patients with and without pyelonephritis.

Unresolved sign	n	Pyelonephritis present	Persistence time in patients with pyelonephritis	Persistence time in patients without pyelonephritis
Striated nephrogram	28	5 (18%)	56 days	91 days
Parenchymal edema	51	10 (20%)	124 days	89 days
Patchy hypodensities	73	17 (23%)	115 days	95 days
Perinephric fat stranding	133	36 (27%)	156 days	122 days
Smooth urothelial thickening	50	17 (34%)	181 days	111 days
Global assessment	37	26 (70%)	101 days	105 days

## Discussion

The time course of resolution of pyelonephritis based on changes in CT findings has not been extensively characterized in the existing literature. Compared with prior studies evaluating MRI or radionuclide imaging, the present investigation specifically examined CT findings, the most commonly used cross-sectional imaging modality in adults with complicated pyelonephritis. Furthermore, the study evaluated the temporal behavior of individual CT findings rather than relying solely on global imaging assessments. This approach provides practical information that may assist radiologists and clinicians in interpreting persistent abnormalities encountered during routine clinical care.

As important as these findings are, we also noted, as shown in Table 3, that despite the clinical resolution of the pyelonephritis, the CT signs consistent with persistence were present for 105 days on average. This information may impact practice, as the low specificity of the signs or the global diagnosis can potentially lead to diagnostic and therapeutic interventions in people without pyelonephritis.

Imaging is not routinely indicated in uncomplicated renal infections, as clinical signs and laboratory data are generally sufficient for making a diagnosis. However, imaging plays a crucial role in specific situations, including immunocompromised patients, treatment non-responders, equivocal clinical diagnosis, evaluation of existing anatomical anomalies, and assessment of disease extent [11]. Unfortunately, histologic specimens of the renal parenchyma in patients with pyelonephritis are seldom obtained, limiting the interpretation of the associated CT signs [9].

While both unenhanced and contrast-enhanced CT can identify urolithiasis, perinephric fluid, renal swelling, and hydronephrosis, only contrast-enhanced CT reliably demonstrates the parenchymal enhancement abnormalities that are characteristic of pyelonephritis. The American College of Radiology notes that in patients without a history of renal stones, the benefit of adding unenhanced CT to contrast-enhanced CT is negligible, as the contrast-enhanced phase alone achieved 96-99% accuracy for detecting urolithiasis in addition to its superior performance for diagnosing pyelonephritis [3]. The signs explored by our radiologists were previously found in 96% of 189 patients with the diagnosis of urinary tract infection, 62% of whom had two or more signs.

A study using MRI to assess the resolution of imaging among patients with acute pyelonephritis showed that, in 30 women with full clinical resolution one month after the diagnosis, 43% showed image-based resolution at one month, and 74% showed resolution after three months [16]. The more rapid apparent recovery observed in that study compared with our results may reflect differences in imaging modality, imaging criteria, patient selection, or interpretation thresholds. Diffusion-weighted MRI abnormalities may normalize differently than CT findings, such as perinephric fat stranding or urothelial thickening. Additionally, the MRI study included only women with complete clinical recovery and used prospectively scheduled follow-up examinations, factors that may have contributed to the shorter reported interval to radiologic resolution. These results, together with the cost of MRI, challenge its utility in defining cured cases or interpreting abnormal signs in patients with relapsed symptoms of pyelonephritis, as described in 44% of women who suffered 147 episodes of urinary infections [17].

Radionuclide-based scans have also been studied in patients with acute pyelonephritis. Technetium-99m dimercaptosuccinic acid scanning has detected changes consistent with acute pyelonephritis in 37 (46%) of 81 patients. The signs disappeared within three months in 18 of 24 of those patients who had adequate follow-up [18]. However, the relatively long waiting time after radiotracer injection, prolonged acquisition time, and high radiation dose may outweigh the benefits of this diagnostic test [19].

The main limitation of our study is its retrospective nature. Conducting this study prospectively would have required scheduling follow-up CT scans at predetermined time points, an approach for which there is no currently clear clinical indication. Moreover, such a strategy would likely incur prohibitive costs and expose patients to potentially unacceptable risks from repeated radiation and contrast administration [20]. Follow-up CT examinations were obtained for a variety of clinical indications, including persistent symptoms, evaluation for complications, or unrelated clinical presentations. Because the timing of these studies was not standardized, variability in follow-up intervals likely contributed to the broad range of observed TTR values and represents a risk for selection bias. Furthermore, the inability to reliably distinguish persistent imaging abnormalities from recurrent episodes of pyelonephritis in all patients in this setting, rather than following a predefined research protocol, meant that detailed documentation of interval symptomatology was not uniformly available. Consequently, some persistent abnormalities may have reflected recurrent infection rather than delayed radiologic resolution. This limitation likely biased our estimates toward longer TTR values and should be considered when interpreting the results.

While the use of three radiologists and calculation of the inter-rater agreement is one of the study’s strengths, the agreement is overall no more than fair, which limits the generalizability of our findings. One study that addressed the ability to detect radiologic signs of pyelonephritis showed a moderate inter-rater agreement between one board-certified emergency physician and one board-certified internist using signs different than the ones we sought. This design makes it impossible to predict how a third reader, or radiologists’ evaluations, would have modified these results [4]. In another study, two abdominal radiologists with three and five years of experience found a high global inter-rater diagnostic agreement; however, it is unclear how a third reader would have modified these results [21].

Another limitation relates to case selection. Although there is broad agreement in the literature regarding the clinical diagnosis of pyelonephritis, other conditions, such as renal infarction, may present with similar defining features, leading to potential misclassification [22]. In addition, pyuria in the setting of a negative urine culture has been reported in up to 15% of patients with infections outside the urinary tract, which may further confound the diagnosis and result in an erroneous attribution to pyelonephritis [11]. Therefore, although diagnostic certainty cannot be fully assured for all patients included in our database, these considerations reflect real-world clinical practice and thus support the external validity of our signs. Moreover, the absence of an independent control cohort further limits the validity of the sensitivity, specificity, and clinical resolution comparisons.

We opted not to consider in the TTR the patients with or without a clinical definition of pyelonephritis whose CT signs never resolved. Instead, we presented separately the duration of signs in Table 3, which are, for the most part, shorter than those presented in Table 2. Although including these cases in this calculation would have resulted in a shorter overall TTR, we censored these observations because we could not determine whether the abnormalities persisted beyond the one-year follow-up period [23]. Regarding the cases with clinical pyelonephritis but unresolved CT signs, we could not determine whether clinical resolution occurred between episodes. Knowing this information could have skewed the results toward a shorter TTR. Finally, as this study reflects the experience of a single hospital, the generalizability of the signs may be limited.

## Conclusions

CT signs supportive of the diagnosis of pyelonephritis may persist for months and, in our series, frequently remained unresolved even when clinical and laboratory data suggested complete resolution. Awareness of this observation may help clinicians in interpreting persistent imaging abnormalities within the appropriate clinical context and may prevent potentially unnecessary investigations and treatments in patients without evidence of ongoing infection.
